# Phase Ib Trial of Phenformin in Patients with V600-mutated Melanoma Receiving Dabrafenib and Trametinib

**DOI:** 10.1158/2767-9764.CRC-23-0296

**Published:** 2023-12-04

**Authors:** Paul B. Chapman, Mark Klang, Michael A. Postow, Alexander Noor Shoushtari, Ryan J. Sullivan, Jedd D. Wolchok, Taha Merghoub, Sadna Budhu, Phillip Wong, Margaret K. Callahan, Bin Zheng, Jonathan Zippin

**Affiliations:** 1 Department of Medicine, Weill Cornell Medicine, New York, New York.; 2 Weill Cornell Medical College, New York, New York.; 3 Research Pharmacy, Memorial Sloan Kettering Cancer Center, New York, New York.; 4 Cutaneous Biology Research Center, Massachusetts General Hospital and Harvard Medical School, Boston, Massachusetts.; 5 Ludwig Institute for Cancer Research, New York, New York.

## Abstract

**Purpose:**

Preclinical studies show that activation of AMP kinase by phenformin can augment the cytotoxic effect and RAF inhibitors in BRAF V600-mutated melanoma. We conducted a phase Ib dose-escalation trial of phenformin with standard dose dabrafenib/trametinib in patients with metastatic BRAF V600-mutated melanoma.

**Experimental Design:**

We used a 3+3 dose-escalation design which explored phenformin doses between 50 and 200 mg twice daily. Patients also received standard dose dabrafenib/trametinib. We measured phenformin pharmacokinetics and assessed the effect of treatment on circulating myeloid-derived suppressor cells (MDSC).

**Results:**

A total of 18 patients were treated at dose levels ranging from 50 to 200 mg twice daily. The planned dose-escalation phase had to be cancelled because of the COVID 19 pandemic. The most common toxicities were nausea/vomiting; there were two cases of reversible lactic acidosis. Responses were seen in 10 of 18 patients overall (56%) and in 2 of 8 patients who had received prior therapy with RAF inhibitor. Pharmacokinetic data confirmed drug bioavailability. MDSCs were measured in 7 patients treated at the highest dose levels and showed MDSC levels declined on study drug in 6 of 7 patients.

**Conclusions:**

We identified the recommended phase II dose of phenformin as 50 mg twice daily when administered with dabrafenib/trametinib, although some patients will require short drug holidays. We observed a decrease in MDSCs, as predicted by preclinical studies, and may enhance immune recognition of melanoma cells.

**Significance:**

This is the first trial using phenformin in combination with RAF/MEK inhibition in patients with BRAF V600-mutated melanoma. This is a novel strategy, based on preclinical data, to increase pAMPK while blocking the MAPK pathway in melanoma. Our data provide justification and a recommended dose for a phase II trial.

## Introduction

Treatment of BRAF V600-mutated melanomas with a RAF inhibitor, such as dabrafenib, results in rapid adaptations including loss of MAPK pathway feedback inhibition (1, 2) and an increase in oxidative phosphorylation (oxphos; refs. 3–5). This may explain the persistence of melanoma despite RAF/MEK inhibition in the clinical setting allowing the emergence of resistant clones over time. Strategies to block oxphos are predicted to enhance the efficacy of RAF inhibitors. Biguanides, such as metformin and phenformin, inhibit mitochondrial complex I of the electron transport chain and thereby decrease oxphos (6, 7). This leads to an increase in the intracellular AMP:ATP ratio, and phosphorylation and activation of AMP-activated protein kinase (AMPK). AMPK negatively modulates enzymes and regulators that are critical for tumor cell growth such as mTOR. Another action of AMPK is the phosphorylation of BRAF on serine 729 in cells with wild-type BRAF (8, 9). This phosphorylation promotes the association of BRAF with 14-3-3 proteins and disrupts its interaction with the KSR1 scaffolding protein, thereby blocking BRAF signaling to the MAP kinase pathway (8, 9). We therefore envision multiple mechanisms by which biguanides might enhance the effect of RAF inhibitors on BRAF V600-mutated melanoma. Supporting this, phenformin enhances the antitumor effects of RAF inhibitors in BRAF V600E melanoma mouse models and has antimelanoma effects even in cell lines resistant to vemurafenib (9). An important observation was that metformin does not have this effect, probably because metformin relies on organic cation transporters, which are not expressed on melanoma cells (9, 10), to enter cells. Phenformin does not require these transporters. This likely explains why metformin had no effect when tested in a clinical trial in patients with melanoma (11).

Phenformin can also affect the tumor immune microenvironment. Recently, we demonstrated that phenformin selectively inhibits myeloid-derived suppressor cells (MDSC) in mice by inducing reactive oxygen species (12). Thus, phenformin has direct inhibitory effects on melanoma and may enhance the ability of the immune system to attack the tumor cells. With these findings in mind, we reasoned that combining phenformin with a RAF inhibitor would lead to enhanced responses in the treatment of patients with BRAF V600-mutated melanoma and could overcome resistance to the RAF inhibitor.

Phenformin, typically at doses of 50–100 mg/day (13–15), was used for the treatment of type 2 diabetes. In 1978, the FDA took it off the market in the United States (16) because it was associated with rare cases of lactic acidosis which was fatal in about 50% of cases, although many of the cases occurred in patients with poorly-controlled diabetes with significant renal and/or hepatic dysfunction. Given the known toxicities of phenformin, including lactic acidosis, we sought to establish the safety of combining phenformin with a RAF inhibitor/MEK inhibitor combination such as dabrafenib/trametinib. We also tested the hypothesis that phenformin would decrease peripheral MDSCs.

## Materials and Methods

### Phenformin

Bulk phenformin was provided by the Division of Cancer Treatment and Diagnosis of the NCI under a materials transfer agreement. Phenformin was encapsulated at the Pharmaceutical Product Facility at Memorial Sloan Kettering Cancer Center (MSKCC) under cGMP procedures. The final product was evaluated for accuracy using UV-high-pressure liquid chromatography (HPLC) in accordance with USP <621>, uniformity of dosage USP <905>, dissolution USP <711>, water content (Karl Fischer) USP <921> as well as analysis for impurities in accordance with USP <232>. These tests were conducted after encapsulation and at 3, 6, 12, 24, and 36 months thereafter. The 50 mg Phenformin HCl (42.5 mg phenformin) capsules contained the following excipients: Microcrystalline Cellulose NF, Sorbitol Powder NF, Sodium Croscarmellose NF, and were encapsulated into Coni-Snap Hard Gelatin Capsule-size 3. These capsules were packaged into Rexam L-60 Screw-Loc Clear-Vu vials (13-dram vial) with child resistant closure. Each vial had a security foil tape. These phenformin capsules were used under an IND 130924 granted by the FDA.

### Clinical Trial Design

In this phase Ib trial, patients with metastatic or stage III inoperable BRAF V600-mutated melanoma were treated with standard dose dabrafenib (150 mg orally twice daily), trametinib (2 mg orally every day) plus phenformin at escalating dose levels. The first cohort received 50 mg orally twice daily of phenformin. Subsequent cohorts were to be treated at 100 mg twice daily, 200 mg twice daily, 300 mg twice daily according to a standard 3+3 dose-escalation design. Cohorts could be accrued to intermediate dose levels (e.g., 150 mg twice daily) based on toxicities observed. Patients could be de-escalated a maximum of two dose levels if necessary for toxicity. Toxicities were graded using Common Terminology Criteria for Adverse Events (CTCAE) 4.0. Dose-limiting toxicities (DLT) were defined as lactic acidosis, any grade 2 neurologic toxicity, most grade 3 toxicities (except transient fatigue, nausea/vomiting, or diarrhea, asymptomatic laboratory abnormalities), or any grade 4 toxicity. Additional toxicities not listed were considered DLTs at the discretion of the principal investigator. Once a recommended phase II dose (RP2D) was identified, we planned to accrue 10 additional patients to the RP2D in a dose-expansion cohort. Clinical responses were assessed using RECIST 1.1.

Patients were eligible if they had stage IV (or inoperable stage III) melanoma with a BRAF V600 mutation. Patients had to be at least 18 years old, have an Eastern Cooperative Oncology Group performance status of at least 2, and have adequate organ and marrow function. In the dose-escalation phase, prior therapy with RAF inhibitors was allowed. In the 10-patient dose-expansion cohort, patients had to be RAF inhibitor-naïve. Aside from standard exclusion criteria, other exclusion criteria included: brain metastases unless asymptomatic and stable for at least 6 weeks on the equivalent of 2 mg/day of dexamethasone or less; type 1 or 2 diabetes, or Hgb A1c >6.5% (the Hgb A1c exclusion was removed in later amendments); concurrent use of hypoglycemic agents; chronic liver or renal disease; known G6PD deficiency; and QTc interval >500 ms unless a bundle branch block was present.

The protocol was approved by the Institutional Review Boards at MSKCC and Massachusetts General Hospital. All patients provided written informed consent prior to participating on this trial.

### Pharmacokinetics

Pharmacokinetics of phenformin has been studied previously (17–19). We conducted sparse pharmacokinetic sampling to confirm bioavailability of our drug product and to measure steady state concentrations. Plasma samples were collected pretreatment, cycle 1 day 8, day 1 of each subsequent 28-day cycle, and off study. Plasma phenformin was measured by MRI Global Inc using a validated, GLP-compliant, HPLC/MS-MS assay.

### Phamacodynamic Studies

We conducted several exploratory pharmacodynamic studies.

#### MDSC Flow Panel

Peripheral blood mononuclear cells (PBMC) were collected pretreatment, cycle 1 day 8, and day 1 of each subsequent 28-day cycle and cryopreserved until needed. Once thawed, PBMC samples were stained with a fixable Aqua viability dye (Invitrogen) and a cocktail of antibodies to the following surface markers: CD14-PerCP-Cy5.5 (BD Biosciences, M5E2), HLA-DR-ECD (Beckman Coulter, Immu-357), Lineage cocktail – CD3/CD16/CD19/CD20/CD56-FITC (BD Biosciences, SK7/3G8/SJ2 5C1/L27/NC AM16.2). Stained cells were acquired on a BD Biosciences LSRFortessa and analyzed using FlowJo software (FlowJo, LLC). CD14^+^HLA-DR^lo^ monocytic MDSC frequencies were derived objectively by gating on live, lineage-negative CD14^+^ monocytes and exporting this population to a computational algorithm based on the monocyte HLA-DR mean fluorescence intensity coefficient of variation and interpolation from MDSC frequencies obtained from the HLA-DR CV spread of Lin^−^ CD14^+^ monocytes of healthy human donors (20, 21).

#### Phosphorylated AMPK Quantification

Banked PBMC samples were stained with a fixable viability dye (Zombie NIR, Invitrogen) and a cocktail of surface markers to identify T-cell populations. After surface stain, cells were fixed and permeabilized using the Foxp3 Transcription Factor Staining Buffer Set (eBioscience). Cells were then stained intracellularly using a polyclonal antibody against phosphorylated AMPK (p-AMPK) alpha-1/2 (Thr172, Thr183; Thermo Fisher Scientific) followed by an Alexa488-conjugated secondary antibody. Stained cells were acquired on a 5-laser Cytek Aurora and analyzed using FlowJo software (FlowJo, LLC).

The data generated in this study are available upon request from the corresponding author.

## Results

### Patient Characteristics

We accrued 18 patients between March 2017 through December 2019. The timing of the dose expansion coincided with the COVID-19 pandemic, limiting accrual and, as a result, only 2 of the 10 planned patients for expansion were accrued. The demographics of these 18 patients are shown in Table 1. The representativeness of study participants is shown in Supplementary Table S1. There were 13 men and 5 women. The median age was 56.5 (range, 36–73). The BRAF mutation was V600E in 14 of the patients and V600K in 4 patients. The stage of melanoma included unresectable stage III (*N* = 5), stage M1b (*N* = 2), M1c (*N* = 9), and M1d (*N* = 2). Eight of the 16 patients in the dose-escalation phase and both patients in the dose-expansion phase had received no prior RAF inhibitor therapy.

**TABLE 1:** Patient demographics

	Phenformin dose level				
	50 mg	100 mg^a^	150 mg	200 mg	All patients
No. of patients	6	5	4	3	18
Gender (M/F)	4/2	3/2	3/1	3/0	13/5
Median age (range)	50 (44–59)	56 (47–68)	59 (26–73)	60 (37–62)	56.5 (36–73)
Melanoma stage					
III	1	2	2	0	5
IVA	0	0	0	0	0
IVB	1	1	0	0	2
IVC	3	2	2	2	9
IVD	1	0	0	1	2
BRAF V600 mutation (E/K)	5/1	3/2	4/0	2/1	14/4
Prior therapy					
RAF targeted therapy	4	2	2	1	9
Checkpoint inhibitor therapy	6	5	4	2	17
No prior therapy	0	0	0	1	1

^a^Includes 2 patients treated in the dose-expansion cohort.

### Dose Levels

The first 6 patients were treated at 50 mg twice daily. We then escalated to 100 mg twice daily and treated 3 more patients. We then escalated to 200 mg twice daily but found this dose level to be poorly tolerated in all 3 patients due to nausea, vomiting, and fatigue. We then accrued 4 patients at an intermediate dose of 150 mg twice daily. This dose level was also found to be poorly tolerated. On the basis of this, we settled on 100 mg twice daily as the dose for the dose-expansion phase and accrued 2 patients in the expansion cohort at 100 mg twice daily before the COVID-19 pandemic forced us to halt accrual.

### Toxicity

The treatment-related adverse events (AE) thought to be at least possibly related to study treatment are recorded in Table 2. Each dot indicates the most serious CTCAE grade toxicity for a unique patient; a red dot indicates toxicities that were deemed dose-limiting. The most common toxicities were gastrointestinal (GI)—nausea, vomiting, anorexia, and elevations of transaminases and alkaline phosphatase—accounting for nine DLTs.

**TABLE 2:**
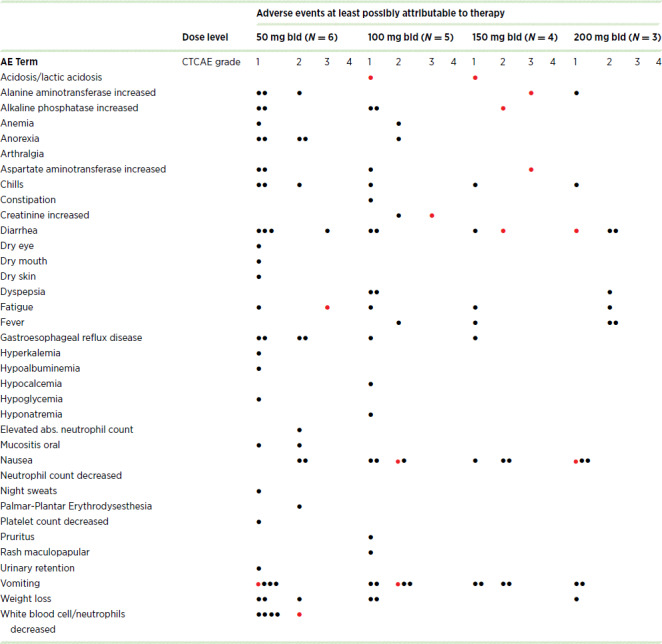
AEs attributable to study drug

NOTE: Each dot represents a patient. If a patient had an adverse event multiple times, only the worst grade event was scored. Red dots indicate adverse events that were considered dose-limiting.

Abbreviation: bid, twice daily.

In the first dose level (50 mg twice daily), 1 patient developed neutropenia that qualified as a DLT and required a total of 6 patients to be treated. Three patients were then treated at 100 mg twice daily without a DLT. The next 3 patients were treated at 200 mg twice daily but this was not tolerable due to GI toxicities. Supplementary Table S2 provides a list of DLTs at each dose level. We assessed the tolerability of the two lowest dose levels based on the need to hold phenformin at least 3 days or the need to reduce the dose (Supplementary Fig. S1). The median time to such an event was 97 days for patients treated at 50 mg twice daily and 28 days for patients treated at 100 mg twice daily.

We observed two cases of anion-gap acidosis. Patient 13 (150 mg twice daily dose level) presented for day 8 of the first cycle with nausea and vomiting. The patient was found to have an anion gap and mildly elevated creatinine. He was admitted, treated with intravenous fluids and anti-nausea drugs and was discharged 2 days later fully recovered. Patient 18 (100 mg twice daily dose level) developed fever, nausea, and vomiting (cycle 1 day 27). She was admitted to a local hospital with elevated creatinine and elevated lactic acid. She was treated with intravenous fluids and after 9 days, she was discharged fully recovered.

Constitutional/inflammatory AEs as well as skin/mucosal events were also observed, presumably due primarily to dabrafenib/trametinib, but constituted only one DLT (fatigue). We also saw hematologic AEs at the lower dose levels including 1 DLT (neutropenia). It is unclear why these AEs were not seen at the higher dose levels, but it could be related to the fact that patients on the lower dose levels were on treatment longer.

Because many studies with metformin, but not all, have reported weight loss, we assessed changes in body weight in our patients on phenformin. In almost all patients, body weight decreased on study (Supplementary Fig. S2). The median weight loss at week 8 was 3.7% (range, 10% loss to 5.1% weight gain) among the 13 patients who were on treatment for at least 8 weeks. We cannot rule out the possibility that some of the weight loss observed was due to transient anorexia.

At the completion of the dose-escalation phase (*N* = 16 patients), we settled on 100 mg twice daily as the dose for further exploration in the dose-expansion cohort. We were only able to accrue 2 patients to this cohort before closing the trial. However, these patients experienced nausea/vomiting and increased creatinine. One of these patients developed lactic acidosis, the other hyponatremia. While all the AEs were reversible, this experience indicates that GI toxicities constitute the primary DLT of this combination therapy.

### Antitumor Responses

**FIGURE 1:**
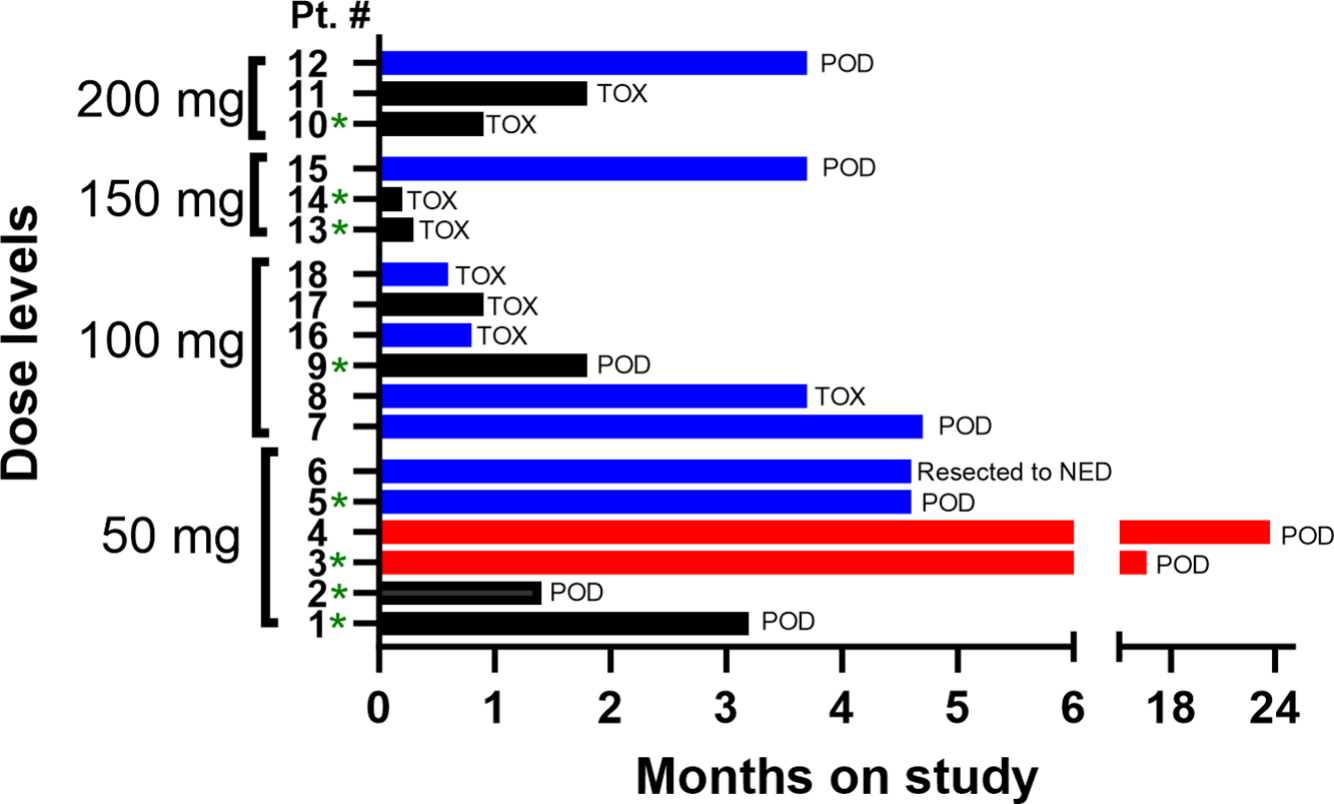
Swimmer plot showing time on study. Patients are grouped by dose level. Red bars indicate patients who had a CR; blue bars are patients with a PR. Black bars indicate patients with no response. Patients who had received prior treatment with RAF inhibitors are indicated with an asterisk. The reason for treatment discontinuation is indicated to the right of each bar. POD, progression of disease; TOX, toxicity. Patient 6 underwent complete resection of residual melanoma and was then taken off study.

Of the 18 patients, there were eight partial responses (PR) and two complete responses (CR; Fig. 1). Responses were observed in 2 of 8 patients who had received prior therapy with RAF inhibitors. One of these patients had a CR that lasted more than a year. This patient had been treated with adjuvant dabrafenib 4 years earlier. The patient with prior RAF inhibitor therapy who experienced a PR had progressed on dabrafenib/trametinib 8 months earlier. There were 8/10 (80%) responders among the RAF inhibitor-naïve patients. One of these patients had a CR that lasted 2 years. Patients 16 and 18 were on phenformin for only 3.5 and 2.5 weeks, respectively, due to toxicity but remained on dabrafenib/trametinib. Each had a PR when assessed at week 7. We acknowledge that this is a small number of patients and that some responding patients were on phenformin for only part of the first treatment cycle, but the high response rate overall, and the observation of responses among patients previously treated with RAF inhibitors, is consistent with the hypothesis that the addition of phenformin can enhance responses to RAF inhibitors.

### Pharmacokinetics

**FIGURE 2:**
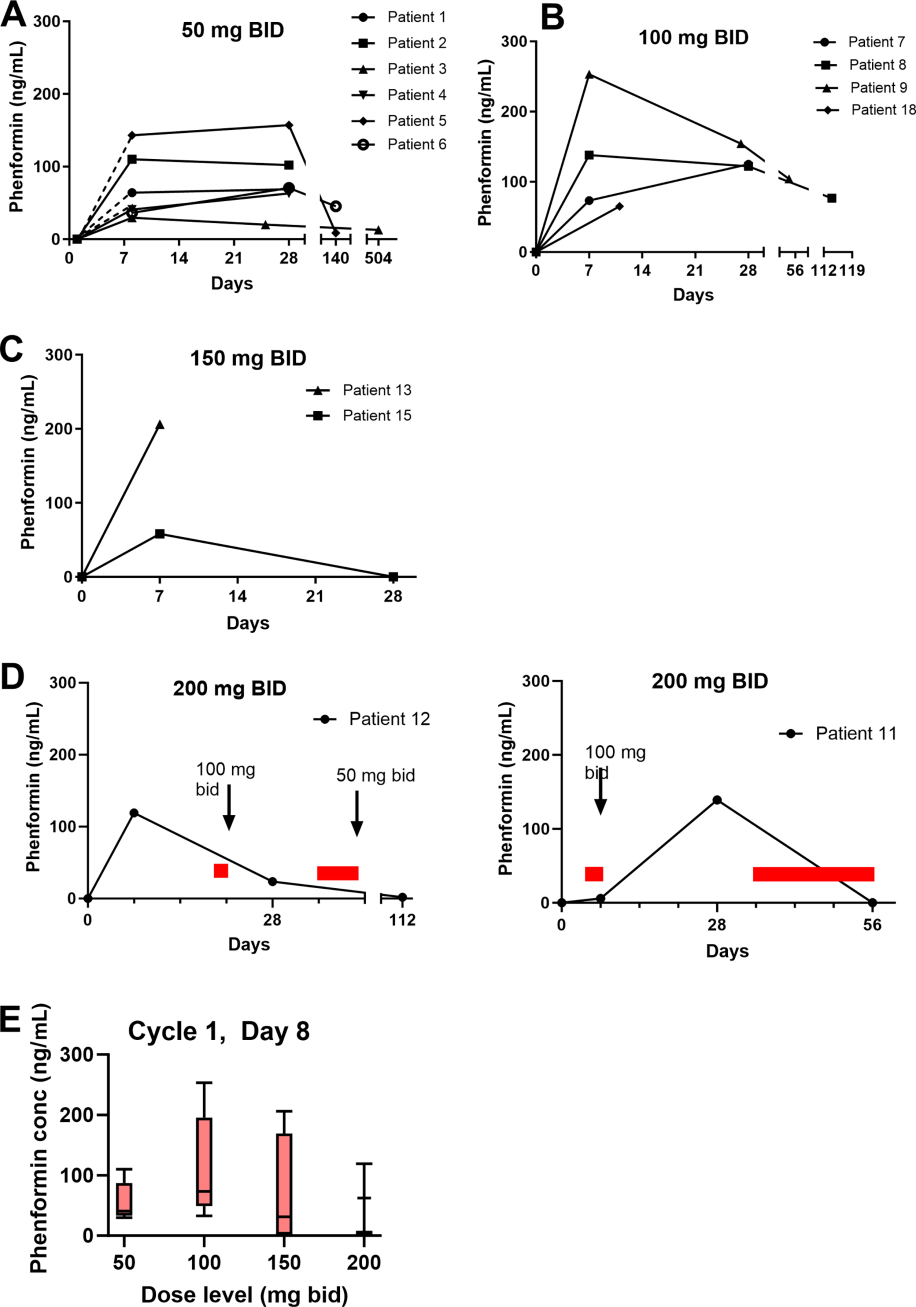
Pharmacokinetics of phenformin. Pharmacokinetic results are shown for the 50 mg bid cohort (**A**), 100 mg bid cohort (**B**), 150 mg bid cohort (**C**), and the 200 mg bid cohort (**D**). Data for patients who missed a substantial number of doses due to toxicity are not shown. For the patients in the 200 mg bid cohort (D), red bars indicate days on which phenformin was held due to toxicity. The arrows indicate dose reductions. **E,** Box and whiskers plot of day 7 phenformin plasma concentrations by dose level. The mean is indicated by the horizontal lines. Each box indicates 25%–75% quartiles; whiskers indicate the range. For the 200 mg dose level, there were data on only 2 patients and so no box is shown. bid, twice daily.

Plasma levels of phenformin were measured pretreatment, day 8, day 28, and at the end of treatment (Fig. 2). Data for patients 10 (200 mg dose level), 14 and 16 (150 mg dose level), and 17 (100 mg dose level) are not included as these patients missed many doses of phenformin due to toxicity. Phenformin levels reached as high as 253.2 ng/mL but when drug was stopped, plasma levels fell rapidly consistent with previous data indicating a plasma half-life of 11–13 hours (19, 22). We compared the day 8 plasma levels across the dose cohorts to examine the relationship between dose and plasma levels (Fig. 2E). This analysis is limited by small patient numbers receiving the higher doses, but it appears that doses over 100 mg twice daily did not result in higher plasma levels at day 8. Patients generally did not tolerate further drug exposure at these dose levels.

### Pharmacodynamics

The proposed mechanism of action for phenformin is an increase in p-AMPK levels. Our intention was to measure changes in PBMC p-AMPK in all patients treated but due to a cell bank malfunction, only samples from patients treated at the highest dose levels (150 and 200 mg twice daily) were available for analysis. Patients treated at these high dose levels experienced frequent AEs requiring phenformin dose interruption, which would be expected to mitigate some of the effects. Despite this, in 3 of 6 patients, there was evidence of p-AMPK increase in PBMC while on phenformin (Supplementary Fig. S3).

**FIGURE 3:**
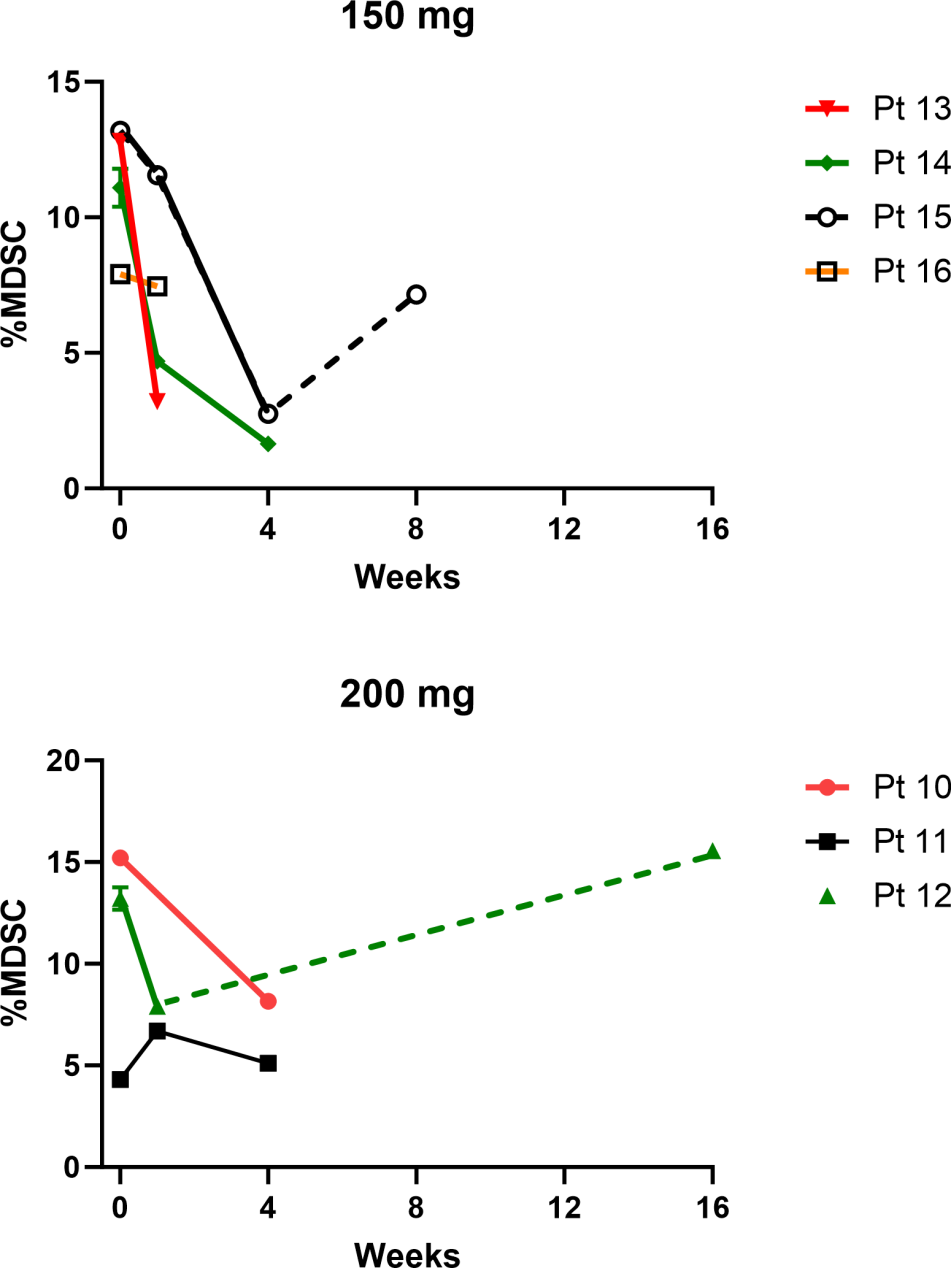
Effect of treatment on percentage of circulating MDSCs derived from flow cytometric analysis of PBMC samples from patients treated at the 150 and 200 mg bid dose level. Dotted lines indicate times when patient was not taking phenformin. bid, twice daily.

We also explored the hypothesis that phenformin would decrease peripheral MDSCs. As noted above, we only had PBMCs from patients treated at the two highest dose levels. Figure 3 shows that for all but one patient (pt 11), numbers of circulating monocytic MDSCs decreased while on phenformin. In patient 11, phenformin was frequently held due to AEs. These data support the hypothesis that phenformin suppresses MDSC. Consistent with this is the observation that in patients 12 and 15, after phenformin was discontinued (dotted lines), monocytic MDSC numbers recovered.

## Discussion

Preclinical observations indicated that biguanide treatment would be expected to enhance the effects of RAF inhibitors on the inhibition of the MAPK pathway. Although metformin penetrates melanoma cells poorly due to lack of expression of organic cation transporters, phenformin can be taken up by melanoma cells in a transporter-independent manner. As such, we were interested in combining phenformin with dabrafenib/trametinib. For this phase Ib trial, we produced and encapsulated phenformin and carried out a dose-escalation trial with dabrafenib/trametinib to establish a RP2D. We had initially planned a dose-expansion cohort in treatment-naïve patients but due to the COVID19 pandemic, we were unable to keep the trial open to accrual. To our knowledge, our trial represents the first human phenformin trial in cancer.

Dose escalation showed that, as expected, the most common AEs and the most common DLTs were GI toxicities—nausea, vomiting, and anorexia. We were especially observant for lactic acidosis because this was the toxicity that led the FDA to remove phenformin from the U.S. market. We found lactic acidosis in 2 patients associated with severe nausea/vomiting. Both patients recovered with discontinuation of phenformin and supportive care. Overall, we recommend 50 mg twice daily as the RP2D for phenformin when combined with dabrafenib/trametinib. However, we recognize that this is based on a small number of patients and that even at this dose, some patients will require a drug holiday. It should be noted that this dose is within the range used previously to treat type 2 diabetes (13). We do not know whether this would be the RP2D for phenformin in combination with other RAF/MEK inhibitor combinations.

We observed 8 responders among 10 treatment-naïve patients (80% response rate). Among the 8 patients who had previously progressed on RAFi/MEKi therapy, there were 2 responders; one of which was a CR. Response rate was a secondary endpoint of this phase I trial and these response rates, although relatively high, could be consistent with the effects of dabrafenib/trametinib alone. We did observe initial signs of pharmacodynamic effects of phenformin such as increased phospho-AMPK in PBMCs, decreased MDSCs, and weight loss in patients. Because of technical limitation and patient specimen restraint, we opted to measure number of circulating MDSCs from patients without investigating their functional status. MDSCs have been suggested to contribute to resistance to targeted therapies, including RAF inhibitor, in preclinical studies (23, 24). Moreover, numbers of circulating monocytic MDSCs have been shown to be correlate with responses to immune checkpoint blockade and patient survival in various cancers (25–27). Our findings regarding the inhibitory effects of phenformin on MDSCs in patients with melanoma confirmed our previous preclinical results from melanoma mouse model (12) and provide rationale for future clinical studies on the combination of immune checkpoint blockage and phenformin.

The toxicity profile of phenformin contrasts with observations from a recent phase I trial with IACS-010759, a potent and selective mitochondrial complex I inhibitor. A dose-escalation trial in patients with acute myelogenous leukemia or other advanced solid tumors was discontinued because of intolerable and dose-limiting neurotoxicity and lactic acidosis (28) which led some to propose a pause in clinical studies of metabolic drugs (29). Our results with phenformin did not prompt such concerns. We believe a randomized trial to quantify the antimelanoma effects of adding phenformin to RAF/MEK inhibitor therapy is justified. A future phase II trial could test the hypothesis that addition of phenformin doubles the median time to progression on dabrafenib/trametinib (12 months improved to 24 months). For such a trial with one-sided type 1 error = 5% and 90% power, we calculate that 96 patients would be needed. We also note that, given the confirmed reduction of MDSCs by phenformin treatment observed in our study and the effects of phenformin on enhancing anti-PD-1 response in mouse models (12), future clinical studies combining phenformin with immune checkpoint blockade could also be considered.

## Supplementary Material

Supplementary Table 1 Representativeness of study participants

Supplementary Table 2 Supplementary Table 2. Dose-limiting toxicities

Supplementary Figure 1 Supplementary Figure 1. Time to first phenformin-related event for patients treated at the 50 mg bid dose level (N=6) and the 100 mg bid dose level (N=5).

Supplementary Figure 2 Supplementary Figure 2. Fold change in body weight of patients by dose level.

Supplementary Figure 3 Supplementary Figure 3. Effect of treatment on pAMPK expression in peripheral blood mononuclear cells.
