# Supplementary material for: Phase Ib Trial of Phenformin in Patients with V600-mutated Melanoma Receiving Dabrafenib and Trametinib
Source: Cancer Res Commun. 2023 Dec 4;3(12):2447–54. doi: 10.1158/2767-9764.CRC-23-0296 (PMC10695100; doi:10.1158/2767-9764.CRC-23-0296)
Supplement: Supplementary Table 1 — Representativeness of study participants [file crc-23-0296-s01.docx]

| **Supplementary Table 1.** Representativeness of Study Participants | |
| --- | --- |
| Cancer type(s)/subtype(s)/stage(s)/condition | Metastatic BRAF V600-mutated melanoma |
| Considerations related to: | |
| Sex | 60% of melanoma cases in US are men and men are more likely than women to develop invasive disease. |
| Age | The median age at the time of melanoma diagnosis is about 65. The average age at metastasis is not well reported. The patients in our trial are somewhat younger. |
| Race/ethnicity | Cutaneous melanoma occurs almost entirely among Caucasian populations. |
| Geography | In this phase I trial conducted at Memorial Sloan Kettering Cancer Center, most patients were from the New York tristate area. |
| Other considerations |  |
| Overall representativeness of this study | The age distribution of our study is similar to the average age distribution of metastatic melanoma.  Men are more likely to have metastatic melanoma. In our study, 72% of our patients were men which is a little higher than the overall population of metastatic melanoma (which is about 60%). |
