# Supplementary material for: Phase Ib Trial of Phenformin in Patients with V600-mutated Melanoma Receiving Dabrafenib and Trametinib
Source: Cancer Res Commun. 2023 Dec 4;3(12):2447–54. doi: 10.1158/2767-9764.CRC-23-0296 (PMC10695100; doi:10.1158/2767-9764.CRC-23-0296)
Supplement: Supplementary Table 2 — Dose-limiting toxicities [file crc-23-0296-s02.docx]

Supplemental Table 2: Dose-limiting toxicities observed at each dose level

| Subject | Dose | AE Term | Grade | Explanation of DLT |
| --- | --- | --- | --- | --- |
| 1 | 50 | Neutrophil count decreased | 3 | Grade 3 lab abnormality that is clinically significant due to dose interruption of Dab/Tram. |
| 2 | 50 | Vomiting | 1 | Resulted in dose reduction and considered a DLT by PI |
| 2 | 50 | Fatigue | 3 | Grade 3 Fatigue persisting longer than 7 days |
| 10 | 200 | Diarrhea | 1 | Resulted in dose reduction and considered a DLT by PI |
| 12 | 200 | Nausea | 1 | Resulted in dose reduction and considered a DLT by PI |
| 12 | 200 | Nausea | 1 | Resulted in dose reduction and considered a DLT by PI |
| 13 | 150 | Lactic Acidosis | 1 | Protocol-defined DLT |
| 15 | 150 | Alanine aminotransferase increased | 3 | Grade 3 lab abnormality that is clinically significant due to dose interruption of Phenformin/Dab/Tram. |
| 15 | 150 | Aspartate aminotransferase increased | 3 | Grade 3 lab abnormality that is clinically significant due to dose interruption of Phenformin/Dab/Tram. |
| 15 | 100 | Fever | 1 | Resulted in drug withdrawal of Phenformin/Dab/Tram.  Considered a DLT by PI |
| 15 | 100 | Alanine aminotransferase increased | 3 | Resulted in drug withdrawal of Phenformin/Dab/Tram.  Considered a DLT by PI |
| 15 | 100 | Aspartate aminotransferase increased | 3 | Resulted in drug withdrawal of Phenformin/Dab/Tram.  Considered a DLT by PI |
| 15 | 100 | Alkaline phosphatase increased | 2 | Resulted in drug withdrawal of Phenformin/Dab/Tram.  Considered a DLT by PI |
| 15 | 100 | Aspartate aminotransferase increased | 2 | Resulted in drug withdrawal of Phenformin/Dab/Tram.  dabrafenib and trametinib attribution is probable |
| 15 | 100 | Aspartate aminotransferase increased | 1 | Resulted in drug withdrawal of Phenformin/Dab/Tram.  dabrafenib and trametinib attribution is probable |
| 15 | 100 | Alanine aminotransferase increased | 1 | Resulted in drug withdrawal of Phenformin/Dab/Tram.  dabrafenib and trametinib attribution is probable |
| 16 | 150 | Diarrhea | 2 | Resulted in dose reduction |
| 17 | 100 | Vomiting | 2 | Resulted in dose reduction Supportive meds given |
| 17 | 100 | Vomiting | 1 | Resulted in drug withdrawal of phenformin Supportive meds given |
| 17 | 100 | Nausea | 1 | Resulted in drug withdrawal of phenformin Supportive meds given |
| 18 | 100 | Creatinine increased | 3 | Resulted in drug withdrawal of phenformin |
| 18 | 100 | Acidosis | 1 | Protocol-defined DLT |
