# Supplementary material for: Phase Ib Trial of Phenformin in Patients with V600-mutated Melanoma Receiving Dabrafenib and Trametinib
Source: Cancer Res Commun. 2023 Dec 4;3(12):2447–54. doi: 10.1158/2767-9764.CRC-23-0296 (PMC10695100; doi:10.1158/2767-9764.CRC-23-0296)
Supplement: Supplementary Figure 1 — Time to first phenformin-related event for patients treated at the 50 mg bid dose level (N=6) and the 100 mg bid dose level (N=5). [file crc-23-0296-s03.pdf]

Supplemental Figure 1

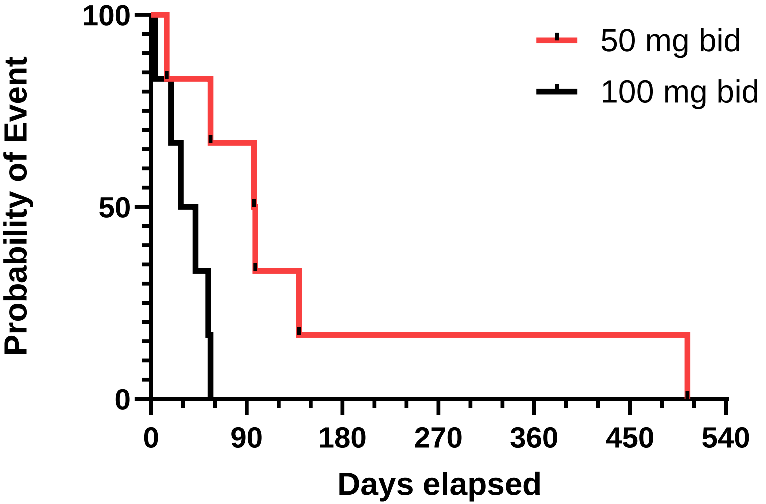

Time to first phenformin-related event for patients treated at the 50 mg bid dose level (N=6) and the 100 mg bid dose level (N=5). An event was defined as the need for phenformin dose interruption for at least 3 days or a dose reduction.
