# Supplementary material for: Phase Ib Trial of Phenformin in Patients with V600-mutated Melanoma Receiving Dabrafenib and Trametinib
Source: Cancer Res Commun. 2023 Dec 4;3(12):2447–54. doi: 10.1158/2767-9764.CRC-23-0296 (PMC10695100; doi:10.1158/2767-9764.CRC-23-0296)
Supplement: Supplementary Figure 2 — Fold change in body weight of patients by dose level. [file crc-23-0296-s04.pdf]

Supplemental Figure 2

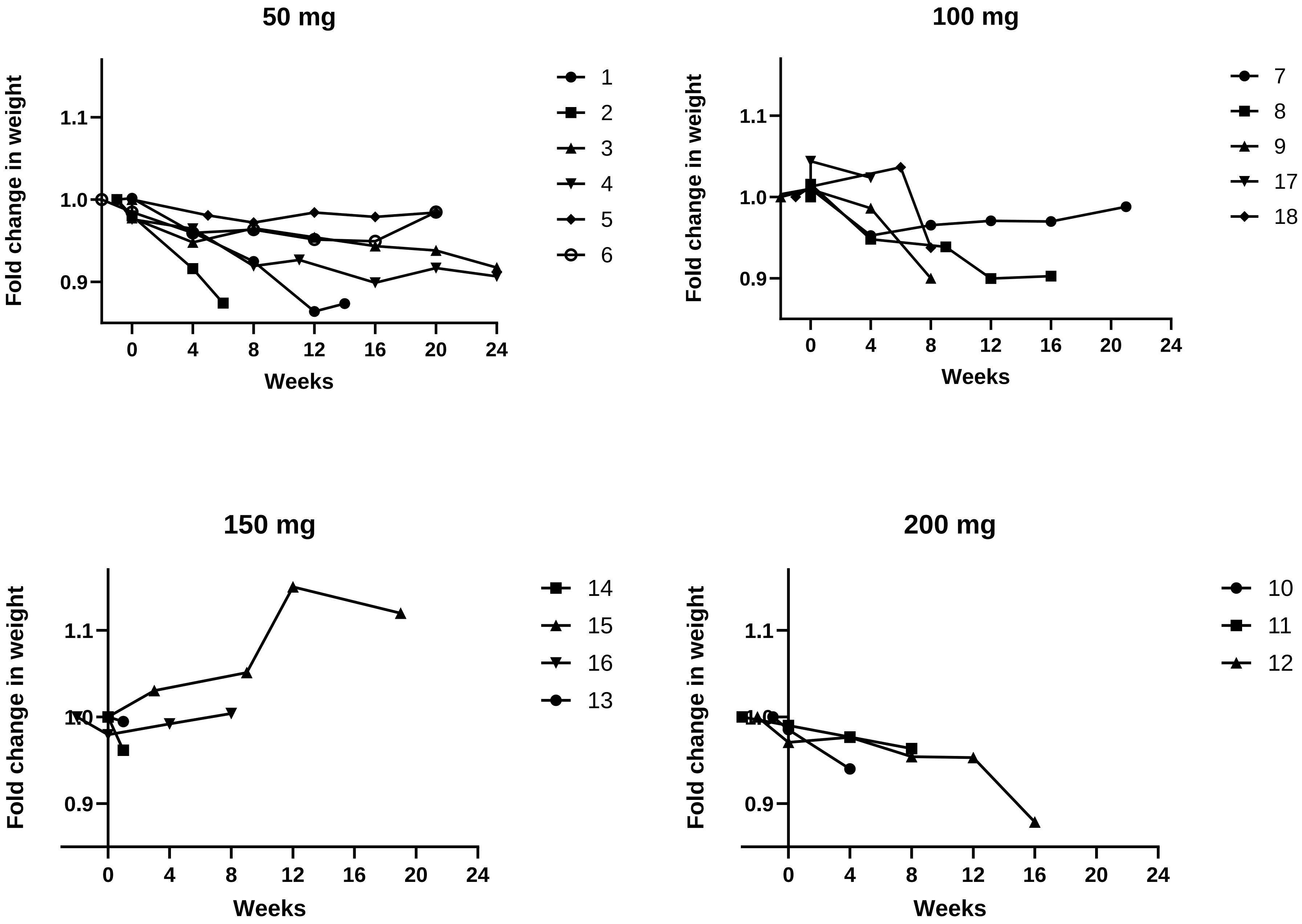

Fold change in body weight of patients by dose level. Patient designation number is as indicated in each graph.
