# Supplementary material for: Phase Ib Trial of Phenformin in Patients with V600-mutated Melanoma Receiving Dabrafenib and Trametinib
Source: Cancer Res Commun. 2023 Dec 4;3(12):2447–54. doi: 10.1158/2767-9764.CRC-23-0296 (PMC10695100; doi:10.1158/2767-9764.CRC-23-0296)
Supplement: Supplementary Figure 3 — Effect of treatment on pAMPK expression in peripheral blood mononuclear cells. [file crc-23-0296-s05.pdf]

Supplemental Figure 3

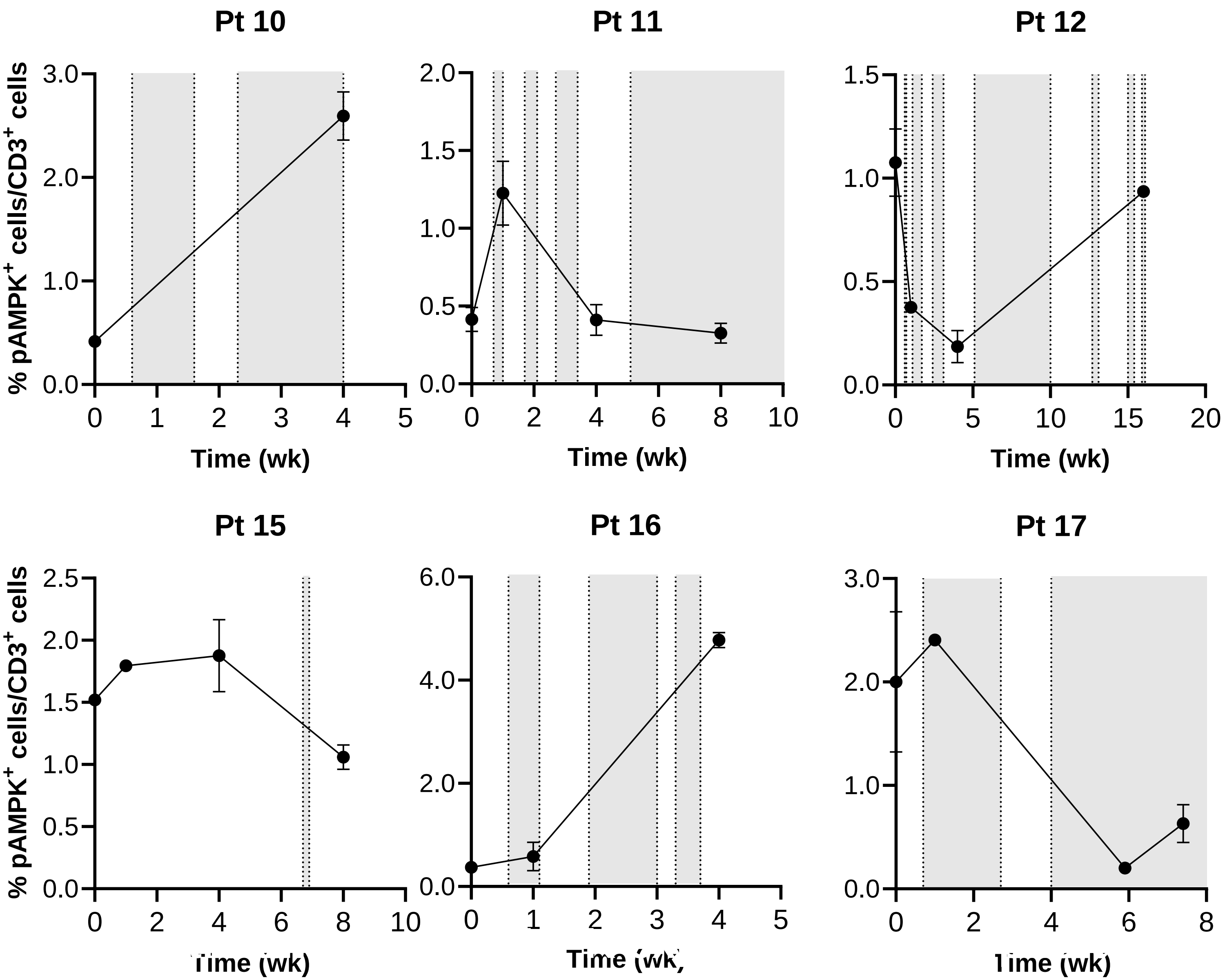

Effect of treatment on pAMPK expression in peripheral blood mononuclear cells. Data shown are the percent of pAMPK-positive cells from patients treated at phenformin dose levels of 200 mg bid (patients 10, 11, 12), 150 mg bid (patients 15 and 16), and 100 mg bid (patient 17). Grey areas indicate times that phenformin was held due to toxicity. Brackets represent standard deviations.
